# A Memory of Professor Robert B. Sim, D. Phil

**DOI:** 10.3390/v13081569

**Published:** 2021-08-09

**Authors:** Chack-Yung Yu

**Affiliations:** Nationwide Children’s Hospital and Department of Pediatrics, The Ohio State University, Columbus, OH 43205, USA; chack-yung.yu@nationwidechildrens.org

Many of us were saddened to learn about the passing of Dr. Robert Sim.

Bob was an exemplary Oxonian scientist with a brilliant quality and originality. He was a kind and humble gentleman, and an outstanding scholar with a high ethical standard, integrity and generosity.

Bob received his tertiary education in Edinburgh, and then Oxford. Prof. Rodney Porter and Prof. Ken Reid were his research mentors and collaborators. He was a senior member of the Medical Research Council (MRC) Immunochemistry Unit at the Oxford Biochemistry Department for over three decades until the Unit’s closure because of Ken’s retirement in 2008 (Figure 1).

Bob’s scientific career reflects the remarkable progress in the field of immune effector mechanisms, particularly the complement system, over the past forty-some years. Bob was a perseverant investigator studying the glycosylations of immunoglobulins, the covalent binding properties of complement C3, the characterization of complement factor H and its related proteins, the complement controlling protein repeats in proteins interacting with C3b or C4b, the mannose binding lectin pathway, and the properties of receptors for processed complement products. The primary focus had been structures and functions. The numbers are the best indicators of Bob’s successes: >300 full-length articles; citations: 29,574; heat index: 97 (as of 31 May 2021, Google Scholar). Interpretation: 97 publications that have been cited more than 97 times in scientific literatures. This is magnificent.

I had the fortune to be a research student at the MRC Immunochemistry Unit between October 1983 and January 1987. At that time, Bob was already a faculty member and his lab neighbored ours, which was headed by Prof. Porter and Dr. Duncan Campbell. Bob’s team was cloning the factor H gene. The Unit was a shining research center that attracted numerous outstanding scientists all over the world. All senior members did bench work and interacted with students and visiting scholars regularly. No matter how busy we were, we had brief coffee/tea breaks twice a day, and those were the best times to ask for inputs! I remember Bob was one of the senior colleagues who read my first research manuscript! Moreover, Edith, Bob’s lifelong spouse, shared with me her then newly improved phenotyping technique, which is still being performed in my laboratory to demonstrate the polymorphism of complement C4.

Many members of the Unit have become our role models in research and life. Bob, you live in our hearts!

## Figures and Tables

**Figure 1:**
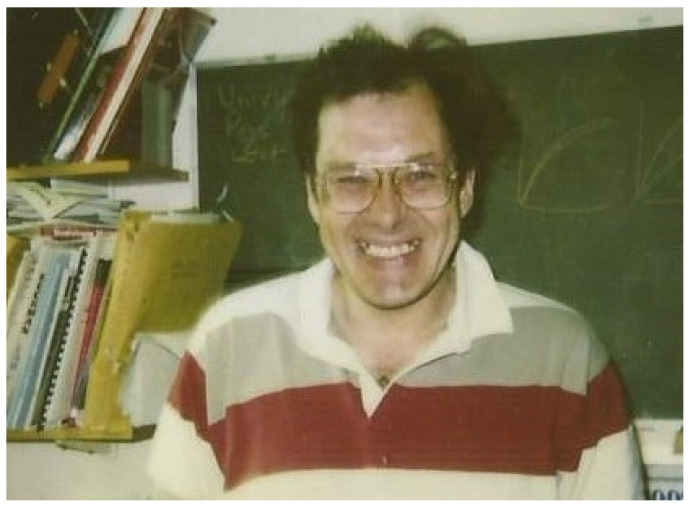
Dr. Robert Sim at work. This photograph was taken in 1992 in Bob’s office at the Rex Richards Building, Oxford. It captures Bob’s joy, energy and enthusiasm in his work (Kindly provided by Bob’s spouse, Professor Edith Sim).

